# Intertwining clonality and resistance: *Staphylococcus aureus* in the antibiotic era

**DOI:** 10.1172/JCI185824

**Published:** 2024-10-01

**Authors:** Henry F. Chambers, Vance G. Fowler

**Affiliations:** 1 Division of Infectious Diseases, UCSF, San Francisco, California, USA.; 2 Division of Infectious Diseases, and; 3 Duke Clinical Research Institute, Duke University, Durham, North Carolina, USA.

*Staphylococcus aureus*, likely the agent of boils, the sixth plague of Egypt (Exodus 9:8–11), is literally a pathogen of biblical proportions. Sir Alexander Ogston first recognized it in 1880 as the most frequent cause of acute abscesses and capable of producing “blood poisoning” with a disease intensity and pace strongly influenced by host factors (1). The situation is very much the same today.

*S. aureus* has conflicting identities as part of the normal human flora, colonizing about a third of the human population, and as a potentially deadly pathogen. Responsible for soft tissue infection, osteoarticular infection, bacteremia, and endocarditis, *S. aureus* is the leading cause of death from bacterial infection in the world (2), while methicillin-resistant *S. aureus* (MRSA) is the leading pathogen-drug combination for death attributable to antimicrobial resistance (AMR) (3). These sobering statistics reflect the ongoing challenges posed by a bacterium that intertwines fitness, pathogenicity, and a remarkable ability to develop resistance to virtually any antibiotic.

## Clones and outbreaks

Another feature of *S. aureus* has been the sudden emergence, expansion, and disappearance of genetically identical hypervirulent clones. Repeatedly, clonality and antibacterial resistance have overlapped. As early as 1954, Knight and Holzer reported that hospital-derived *S. aureus* isolates with group III phage patterns were resistant to multiple antibiotics, demonstrating that antibiotic resistance clustered in specific clones (4). Recognition of these epidemic clones was made possible by the ability to characterize single bacterial strains. Initially, *S. aureus* clone types were characterized by bacteriophage typing on the basis of bacteriophages that lysed the bacteria. Using this technique, a 1954 report described an outbreak of invasive *S. aureus* in neonatal nurseries throughout the United States, Australia, and Europe, consisting primarily of abscesses among the infants, breast abscesses among the nursing mothers, and extended persistence of the clone within families of the infants (4). The causative *S. aureus* strain was resistant to penicillin, streptomycin, and tetracyclines and was lysed by bacteriophages 42B, 47C, 44A, 52, 80, and 81 (5). The 80/81 clone of *S. aureus*, as it came to be known, was ultimately found to be the cause of a global epidemic throughout the 1950s, only to decrease in importance in the 1960s shortly after methicillin was introduced for the treatment of penicillin-resistant *S. aureus*. In the 1980s, new clones of MRSA emerged, all associated with hospital acquisition. By the late 1990s, however, a new phenomenon was reported: MRSA infections occurring in community-dwelling patients with no history of health care contact due to a single bacterial clone that came to be known as USA300. The USA300 epidemic shared many of the features with that of 80/81 two decades before, including a tendency to cause cutaneous abscesses and a high infection rate among pediatric populations. Almost overnight, USA300 became the most common cause of skin and soft tissue infection in the United States (6). In other parts of the world, other distinct strains of MRSA established themselves, including the ST93 hypervirulent clone in Australia (7), and ST398, a clone of MRSA associated with livestock (7). While the rates of MRSA have declined in much of the world, including the United States (8), it continues to cause more multidrug-resistant bacterial infections in the United States than all other bacteria combined (9).

## Antibiotics and resistance

**Figure 1:**
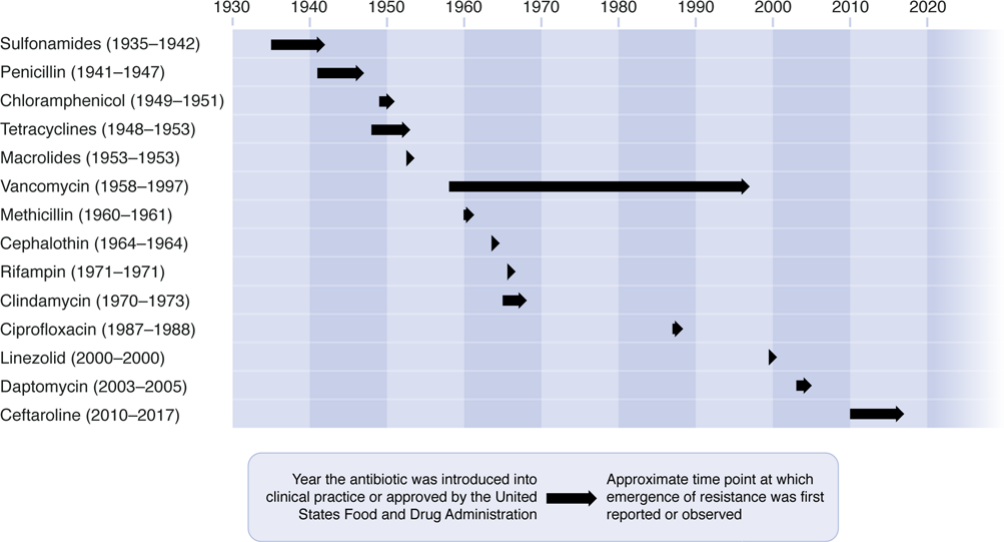
Timeline for the emergence of *S. aureus* resistance upon introduction of antibiotics into clinical practice. Adapted from Stennett et al (29).

*S. aureus* infections in the preantibiotic era were frequently a death sentence, with mortality rates exceeding 80% (10). While the introduction of penicillin into clinical practice in the 1940s revolutionized the treatment of *S. aureus* infections, resistance to penicillin emerged soon thereafter to threaten these improvements (11). Since then, an antibacterial arms race has ensued, with the introduction of new antibiotics inevitably being followed by resistance in *S. aureus* clinical isolates (Figure 1). Resistance to penicillin was due to production of a penicillinase, an enzyme that inactivates the drug by hydrolyzing its β-lactam ring. Although not recognized at the time, the penicillinase that the investigators identified was almost certainly encoded by a gene carried on a horizontally transferable, mobile genetic element. Thus, two paradigms of staphylococcal drug resistance were established from the very beginning: *S. aureus* can develop resistance to virtually any antibiotic, and horizontal gene transfer is a preferred mechanism by which resistance is acquired. Gene transfer may occur by any of three mechanisms: transformation (uptake of extracellular DNA), transduction (phage-mediated DNA transfer), or conjugation (DNA transfer from direct cell-to-cell contact), the latter two being the more common.

*S. aureus* has a vast array of mobile gene elements at its disposal including plasmids, insertion sequences, transposons, integrative and conjugative elements, and gene cassettes such as staphylococcal cassette chromosome (SCC*mec*), which encodes the methicillin-resistance gene *mec* as well as other resistance genes. The emergence of MRSA, which occurred almost immediately upon introduction of methicillin into clinical practice, has been particularly problematic. MRSA exhibits class resistance to almost all β-lactam antibiotics, the preferred drugs for treating staphylococcal infections because of their reliable safety and efficacy. MRSA strains are also often multiple-drug resistant, further limiting treatment options. Vancomycin has long been the mainstay antibiotic to treat MRSA infections. Despite it being the most commonly prescribed intravenous antibiotic in the United States (12), resistance to vancomycin in *S. aureus* was slow to develop. After almost half a century of clinical use, however, low- and high-level forms of resistance to vancomycin emerged in *S. aureus* in 1996 and 2002, respectively. Mechanistically, the two forms differ. Vancomycin-intermediate *S. aureus* (VISA) exhibits low-level resistance to vancomycin by trapping the antibiotic within a thickened peptidoglycan cell wall. Vancomycin-resistant *S. aureus* (VRSA) demonstrates full resistance through acquisition from enterococci of the *vanA* gene cluster, which codes for remodeling of the peptidoglycan precursors (13). Both forms of reduced vancomycin susceptibility in *S. aureus* remain uncommon. While the availability of alternatives to vancomycin such as linezolid, daptomycin, and ceftaroline improved the situation, strains of S. aureus resistant to all of the drugs were quickly encountered in the clinic.

Most recently, several innovative compounds targeting MRSA have progressed to clinical trials, with mixed results. Bacteriophages, a promising but unproven therapy for severe MRSA infection (14), are currently in double-blind, randomized trials. Lysins, a new class of bacteriophage-derived antibacterials, have also advanced to clinical trials. Compared with bacteriophages, recombinant lysins have the advantage of avoiding the emergence of resistance and possible horizontal gene transfer, as well as some of the complexities associated with developing and marketing a virus for pharmaceutical purposes. In a randomized, double-blind, placebo-controlled phase II trial, patients with MRSA bacteremia who received the lysin exebecase in addition to standard antibiotics had a significantly higher clinical success rate at day 14 of treatment (15). Despite this promising result, phase III of the trial was halted for futility (16). The reasons underlying the dramatic differences in the two exebecase trials are unknown but may relate to the small sample size in the phase II trial, exclusion of patients with left-sided endocarditis in phase III, and the fact that the primary efficacy assessment at 14 days may have been too early to allow full resolution of patient symptoms. Similarly, suvratuxomab, a monoclonal antibody targeting α-toxin, showed some promise in preventing *S. aureus* ventilator-associated pneumonia in a phase II trial (17), but the phase III trial has been on voluntary hold since 2022. One bright spot in antibiotic development was the FDA approval in April 2024 of ceftobiprole, an anti-MRSA cephalosporin similar to ceftaroline, for *S. aureus* bacteremia including MRSA (18).

## Colonization and prevention

*S. aureus* asymptomatically colonizes approximately one-third of the population. These individuals are at increased risk of infection with their colonizing *S. aureus* clone (19). As a result, investigators have sought to diminish this risk of *S. aureus* infection by reducing or eliminating colonization. In the 1960s, investigators tested strain interference, in which deliberate colonization with a less virulent strain of *S. aureus* could reduce the risk of infection by interfering with the subsequent acquisition of a more virulent strain. This strategy was utilized to reduce rates of the 80/81 outbreak by colonizing neonates with *S. aureus* 502, a strain thought to be avirulent (20). While the rates of infection with 80/81 declined, work in interference abruptly stopped after an infant died of meningitis caused by the ostensibly avirulent strain. Decolonization has been far more successful, as it was shown to reduce the rates of MRSA infection in nursing homes (21) and intensive care units, in MRSA-colonized patients following discharge (22), and in a regional collaborative of universal decolonization in long-term care facilities (23). A recent double-blind, placebo-controlled phase II study in Thailand found that ingestion of a probiotic containing *Bacillus subtilis* reduced more than 95% of the total *S. aureus* colonizing the study participants without otherwise altering the microbiota (24).

In contrast to the relative success of decolonization, immunotherapeutic approaches (both active and passive) have been failures to date. Vaccines to prevent *S. aureus* infections have failed in clinical trials of patients undergoing hemodialysis, cardiac surgery, or elective spinal surgery (25). Although there are probably multiple reasons for the failure of these trials, neutralization of staphylococcal toxins may ultimately be more effective than the opsonophagocytosis-based strategies they used (25). Furthermore, recent work has shown that, in contrast to vaccinated naive mice, mice previously infected with *S. aureus* were unable to mount a protective antibody response to vaccination with IsdB, producing antibodies that blunted opsonophagocytosis, targeted a nonprotective IsdB domain, and elicited direct antibody competition (26). Development of a *S. aureus* vaccine has been further complicated by the fact that, in one trial, participants who received the IsdB vaccine and subsequently developed *S. aureus* infection were five times more likely to die than placebo recipients who developed *S. aureus* infection (27). This finding was recapitulated experimentally. Mice vaccinated with whole killed *S. aureus* that were subsequently infected with *S. aureus* were significantly more likely to die than unvaccinated, infected mice. Death was due to a CD4 T cell–dependent interferon-γ (IFN) response and could be prevented by inhibiting the IFN response (28). Whether Th1 cells cause a similar deleterious response during vaccine-induced immunity against *S. aureus* infection in humans is unknown but should be considered in future vaccine trials (25). Monoclonal approaches have been similarly unsuccessful.

## Conclusion

Over the past century, *S. aureus* has been characterized by the emergence of new clones and new forms of antibiotic resistance. While development of new antibiotics and, ideally, new antibiotic classes continues to be important, new approaches and strategies will be required to meet future challenges posed by this highly adaptable pathogen.

## Version 1 10/01/2024

Electronic publication
